# Controlled Reduction of Sn^4+^ in the Complex Iodide Cs_2_SnI_6_ with Metallic Gallium

**DOI:** 10.3390/nano13030427

**Published:** 2023-01-20

**Authors:** Shodruz T. Umedov, Anastasia V. Grigorieva, Alexey V. Sobolev, Alexander V. Knotko, Leonid S. Lepnev, Efim A. Kolesnikov, Dmitri O. Charkin, Andrei V. Shevelkov

**Affiliations:** 1 Department of Materials Science, Lomonosov Moscow State University, Leninskie Gory 1/73, 119991 Moscow, Russia; 2 Department of Chemistry, Lomonosov Moscow State University, Leninskie Gory 1/3, 119991 Moscow, Russia; 3 Department of Chemistry, MSU-BIT University, Shenzhen 517182, China; 4 Lebedev Physical Institute of the Russian Academy of Sciences, Leninskiy Prospect 53, 119333 Moscow, Russia

**Keywords:** ampoule reactive sintering, halide perovskite, chemical reduction in melt, ^119^Sn Mössbauer spectroscopy, substitutional solid solutions

## Abstract

Metal gallium as a low-melting solid was applied in a mixture with elemental iodine to substitute tin(IV) in a promising light-harvesting phase of Cs_2_SnI_6_ by a reactive sintering method. The reducing power of gallium was applied to influence the optoelectronic properties of the Cs_2_SnI_6_ phase via partial reduction of tin(IV) and, very likely, substitute partially Sn^4+^ by Ga^3+^. The reduction of Sn^4+^ to Sn^2+^ in the Cs_2_SnI_6_ phase contributes to the switching from *p*-type conductivity to n-type, thereby improving the total concentration and mobility of negative-charge carriers. The phase composition of the samples obtained was studied by X-ray diffraction (XRD) and ^119^Sn Mössbauer spectroscopy (MS). It is shown that the excess of metal gallium in a reaction melt leads to the two-phase product containing Cs_2_SnI_6_ with Sn^4+^ and β-CsSnI_3_ with Sn^2+^. UV–visible absorption spectroscopy shows a high absorption coefficient of the composite material.

## 1. Introduction

Complex tin-based halides [1] with a general formula A^I^M^II^X_3_ (where A is an inorganic or organic cation such as Cs^+^, K^+^, Rb^+^, or CH_3_NH_3_^+^ [2]; M^IV^ = Sn; and X = a halide anion of F^−^, Cl^−^, Br^−^, or I^−^) are most promising as light-harvesting components in modern photovoltaic solar cells (PSCs) as alternatives to lead halides. Recently, the power-conversion efficiency of Pb-based inorganic–organic perovskite PSCs has overrun 25% in single-junction architecture [3]. Such progress of perovskite-solar-cell efficiency is a result of both the chemical design of new light-harvesting compounds and also the evolution of the overall structure of the photovoltaic cell that originated from the architecture of the dye-sensitized solar cell [4] and transformed later to a thin sandwich-like structure with nano-sized functional layers of solid materials [5]. Research of complex halides as new light-harvesting materials gives a chance to minimize significantly the size and make flexible perovskite photovoltaic cells.

It is remarkable that all of the most efficient perovskite solar cells are based on lead-containing light absorbers; however, this raises concerns owing to their high toxicity [6,7]. Despite the progressive increase in the efficiency of lead PSCs, the toxicity of lead requires development of new lead-free analogues with appropriate optical and electrical characteristics. Lately, γ-CsSnI_3_ with the *p*-type conductivity was used in PSCs as a light-harvesting compound; however, the phase has demonstrated rather poor stability to oxidation and hydrolysis [8,9,10,11,12]. Ichiba and Kanatzidis described four polymorphs for CsSnI_3_, including black B-α-CsSnI_3_, black B-β-CsSnI_3_, black B-γ-CsSnI_3_, and yellow (or green) Y-γ-CsSnI_3_ [13,14]. Optical methods have shown that, among these polymorphs, the black metastable B-γ-CsSnI_3_ phase demonstrates metallic conductivity, high hole mobility, rather strong luminescence, and a large optical absorption coefficient [15,16,17], as well as the improved phase stability in a close-packed cell [8,18,19]. The compound is also suitable for PSCs as a hole conductive layer [20] or light-absorber layer [21]. Despite this, instability problems of γ-CsSnI_3_ as a light-harvesting compound in cycling and slow degradation reduce the lifetime and efficiency of such PSCs. The reasons for instability of the CsSnI_3_ phases originate from the high reduction activity of Sn^2+^, which is easily oxidized to Sn^4+^ in moist air and disproportionately converted into Sn^4+^ and Sn^0^ in an inert atmosphere. Some problems with the negative-charge-carrier transfer could also take place.

Recently, Lee et al. [22] suggested the perovskite-like phase of Cs_2_SnI_6_ as a more stable analogue among tin-based lead-free perovskite light harvesters [23,24]. Since that moment, Cs_2_SnI_6_ in PSCs has been explosively developing as a light absorber or semiconductive PSC compound and is attributed to “double perovskites”. Actually, the phase of Cs_2_SnI_6_ with the *Fm*-3*m* space group and the lattice parameter *a* in the range of 11.6276(9) Å [12]—11.65 Å [25,26] is not a “double perovskite” phase but is a complex halide of a K_2_PtCl_6_ structure type. Its transport characteristics are also still under discussion. According to Saparov et al. [27], Cs_2_SnI_6_ is an *n*-type semiconductor with a direct band gap (*E_g_*) of 1.26 [22] or 1.62 eV [28]. Lee et al. [22] have presented Cs_2_SnI_6_ as a *p*-type semiconductor for the HTM layer according to its non-stoichiometry and presence of some Sn^2+^ in Sn^4+^ positions. The air stability of Cs_2_SnI_6_ is not only due to the 4+ oxidation state in this compound but also due to the shorter interatomic distance and stronger covalency of the Sn–I bonds in the [SnI_6_]^2–^ octahedra than in perovskite structure of CsSnI_3_ [27,29].

Doping of Cs_2_SnI_6_ with some other elements leads to the formation of new intrinsic defects and to an increase in concentration of charge carriers, which improves the stability of compounds [30,31,32]. Recently, Lee et al. [33] reported that the partial iodine replacement by bromine (Cs_2_SnI_6−x_Br_x_) using a “sandwich” device-fabrication process (a two-step-solution processing technique) makes it more air stable. Nowadays, Cs_2_SnI_4_Br_2_ is known as the most air-stable compound of the Cs_2_SnI_6_ type [33]. Recently, we showed the possibility of heterovalent substitution of tin by In^III^ in Cs_2_SnI_6_ [34].

In this study, we report the effect of metal gallium as a reducing agent for the complex iodide Cs_2_SnI_6_. We also report here the formation of the substitutional solid solution Cs_2_Sn_1-x_Ga_x_I_6-x_ belonging to the K_2_PtCl_6_ structure type [35,36]. The reduction of Sn^4+^ to Sn^2+^ by metal gallium melt leads to a complex product with varying Sn^2+^ percentage. A general composition of products with Cs_2_SnI_6_-based substitutional solid solution (SS) could be expressed by a general formula of Cs_2_Sn^4+^_1-x_Ga^3+^_x_I_6-x_, where *x_max_* is up to 0.15. For the samples obtained with an excess of reductant (RS), no elemental iodine was added to the ampoules (as shown in SI, Table S1).

XRD and Sn^119^ Mössbauer spectroscopy were applied to investigate the reaction products at different gallium-to-iodine ratios.

Both fundamental and structure-sensitive characteristics of compounds are important for the new materials proposed as light-harvesting compounds. The investigation of Ga-doped (IV) cesium iodostannate as light harvesters included both phase analysis and analysis of the microstructure at the nanolevel.

## 2. Materials and Methods

### 2.1. Materials and Syntheses of RS/SS Compounds

The reactions were carried out in sealed quartz ampoules and evacuated to a pressure of 1.6∙10^−2^ Torr. The reduction samples (RS compounds) (where *x* = 0–0.2) were prepared by grinding cesium iodide (CsI) (SigmaAldrich, St. Louis, MO, USA, 99.9%), tin iodide (SnI_4_), and metallic gallium (Ga) (SigmaAldrich, 99.9999%) with the stoichiometric mass ratios given in Table S1 in Supplementary Materials in an agate mortar. The solid-solution samples of Cs_2_Sn_1−x_Ga_x_I_6−x_ (SS compounds) were prepared in the same way but with the addition of elemental iodine in small excess amounts proportional to the cesium content. Mass ratios are given in Table S2 in Supplementary Materials. The pristine Cs_2_SnI_6_ phase was obtained using the stoichiometric molar ratio of CsI to SnI_4_ (2:1). The mixtures were sealed in preliminary dried-quartz ampoules and heated with a rate of ~0.2 °C/min to 300 °C and then annealed at this temperature for 96 h. The specimens were cooled to room temperature slowly and opened inside a nitrogen-filled glove box. All products were black in color, and irregularly shaped crystals were observed under a microscope. All samples were kept in closed black Ziploc bags in nitrogen and then studied at room temperature in air.

### 2.2. Characterization Methods

X-ray diffraction measurements (XRD) were performed on a Rigaku D/MAX 2500 diffractometer (Rigaku, Tokyo, Japan) equipped with a rotating copper anode (Cu-Kα_1,2_ radiation) and operated at 45 kV and 250 mA from 5 to 80° in 2Ɵ, and the scanning rate was 3° min^−1^ at a step of 0.02°. The experimental data were analyzed using WinXPow (database PDF2) (STOE & Cie, Version 1.07, Darmstadt, Germany) to define the phase composition, whereas for the lattice parameter calculations, Jana 2006 software (ECA-SIG#3/Institute of Physics, Prague, Czech Republic) was applied. The crystal structure of the materials was designed using the VESTA (JP-Minerals, Tsukuba, Japan) and Diamond (Crystal Impact, Bonn, Germany) programs.

Mössbauer spectroscopy experiments were carried out in closed polycarbonate ampoules during 2 days using an original setup equipped with a Ba^119m^SnO_3_ source. Mössbauer spectra at ^119^Sn nuclei were recorded on an MS-1104Em electrodynamic spectrometer operating in a constant-acceleration mode (CJSC Cordon, Rostov-on-Don, Russia). The analysis and model approximation of the spectra were performed with the SpectrRelax software application [37,38].

UV–visible diffuse reflectance spectra were collected using the UV–visible spectrometer Lambda 950 (PerkinElmer, Waltham, MA, USA). Measurements were performed in a spectral range of 200–2000 nm with a step scan of 1 nm at 298 K with a scanning rate of 1 nm/s using quartz glass as a reference. The optical energy band gap (*E_g_*) was acquired using a Tauc plot as a dependence of (αhν)^2^ on energy (hν).

The microstructure of the samples was studied using a scanning electron microscope with the field emission source LEO SUPRA 50VP (LEO Carl Zeiss SMT Ltd., Oberkochen, Germany) with 250 X–2.5k X magnification. The samples were analyzed by X-ray emission microanalysis with an X/MAX X-ray energy dispersive detector (EDX) (Oxford Instruments, High Wycombe, UK).

## 3. Results and Discussion

The SS samples of the estimated composition of Cs_2_Sn^4+^_1−x_Ga^3+^_x_I_6−x_ and RS samples (assuming a composition of Cs_2_Sn^4+^_1−5x_Sn^2+^_3x_Ga^3+^_2x_I_6−8x_) with *x* = 0–0.2 were synthesized by a reactive sintering method in vacuum (in closed ampoules), while almost in all quoted publications various wet-chemistry methods were applied for synthesizing Cs_2_SnI_6_ and other complex iodide materials. The compounds taken in the stoichiometric mass ratios for the SS series are given in Table S1 in Supplementary Materials. Compositions of the RS samples had a lack of elementary iodine and an excess of metal gallium (Table S1, in Supplementary Materials). The weight of each sample was 0.01 g. We observed no traces of the possible reactions between iodine, tin iodide, or metal gallium with quartz ampoules because any reactions were negligible and did not change the stoichiometry of elements during the synthesis.

**Figure 1:**
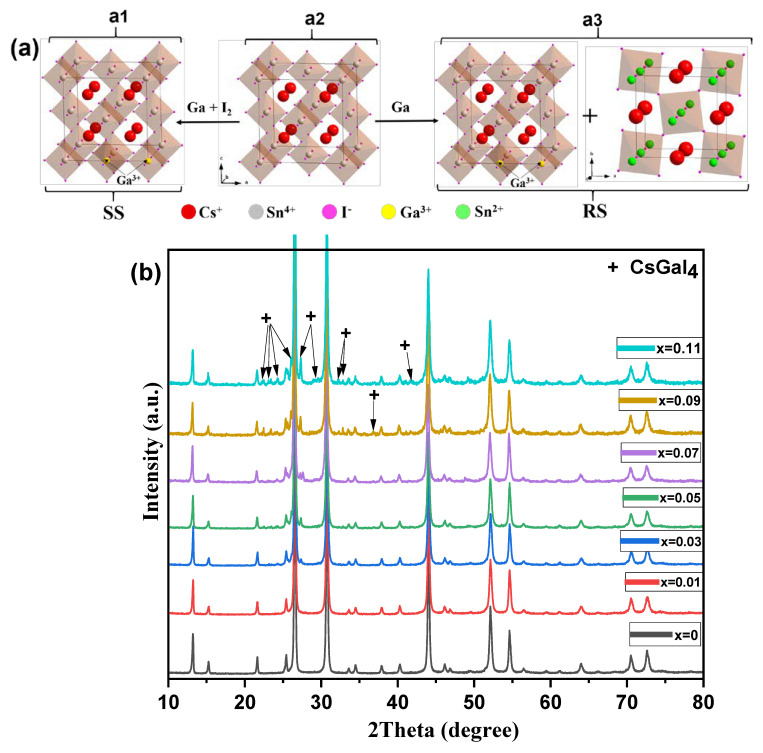

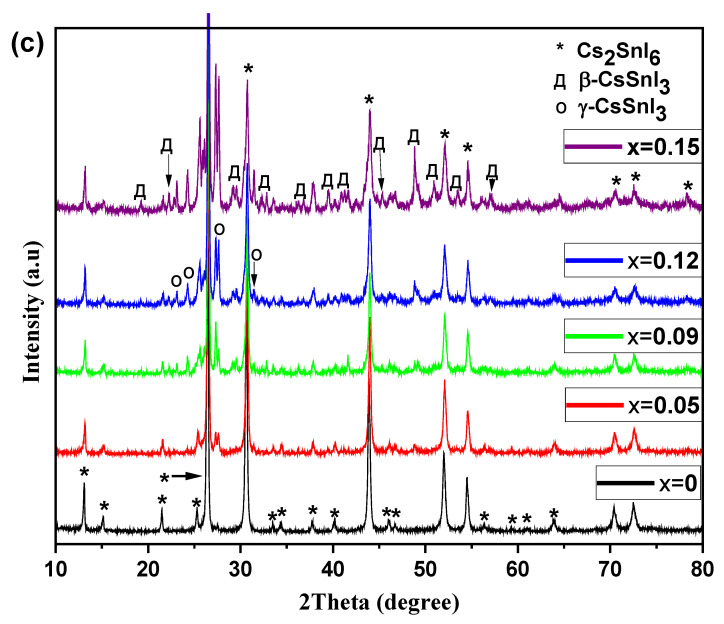
(**a1**) Possible positions of heteroatoms (Ga^3+^) in Cs_2_SnI_6_ perovskite lattice; (**a2**) Cs_2_SnI_6_ unit cell; (**a3**) Cs_2_SnI_6_ reduction result. XRD data for (**b**) Cs_2_SnI_6_-based solid solutions and (**c**) the samples obtained by reduction with the melt of metallic gallium (*x* = 0–0.15). In diffractograms of SS samples, impurity-phase CsGaI_4_—reflections are marked with + symbols—and all unmarked reflections belong to Cs_2_SnI_6_. In diffractograms of RS series, β-CsSnI_3_ peaks are marked with ⊗-symbols, γ-CsSnI_3_ peaks are marked with ο-symbols, and Cs_2_SnI_6_ peaks with the symbol *.

Samples of the SS series were synthesized in small excess amounts of elemental iodine to produce the I-reach materials with the *n*-type conductivity and low deficiencies of the anionic sublattice. We supposed the Sn^4+^ octahedral positions in the Cs_2_SnI_6_ structure to be the most preferable for heterovalent substitution by Ga^3+^ (0.620Å in the octahedral environment), while the reduced tin (Sn^2+^) cation (1.16 Å) is larger significantly than the 0.690 Å of the Sn^4+^ ionic radii, which works against the formation of the substitutional solid solution. We proposed the possible products in the RS series to be Cs_2_SnI_6_-like compounds with Sn^4+^ cations substituted partially by Ga^3+^ or Sn^2+^ cations (Figure 1a).

It is shown experimentally that all characteristic XRD reflections for the SS series (Figure 1) match well to the peaks generated from the crystal data and are consistent with the experimental data for the Cs_2_SnI_6_ phases reported elsewhere [23,39]. For all samples of the SS series, XRD reflections are consistent with the cubic vacancy-ordered perovskite-like structure (Figure 1b). The noticeable shift in the reflection positions to higher or lower 2Ɵ values with increasing Ga/Sn ratio *x* is not observed.

According to Figure 1b, the XRD patterns of two samples related to the SS series (namely, *x* = 0.01 and 0.03) and one related to the pristine Cs_2_SnI_6_ (where *x* = 0) can be regarded as single phase. Contrastingly, the XRD patterns of the RS series (Figure 1c) distinctly show the presence of the β-CsSnI_3_ secondary phase. The most intensive diffraction peaks are associated with reflections of Cs_2_SnI_6_ (PDF2 file #73-330), while the weaker group of peaks is associated with reflections of γ-CsSnI_3_ (PDF2 file #71-1898). The intensities and number of CsSnI_3_-characteristic reflections increase with the percentage of gallium in the samples before the heat treatment (0→0.15). Meanwhile, diffractograms do not show reflections of any iodogallates such as CsGaI_4_ and CsGa_2_I_7_ (Figure 1c).

It is remarkable that for the samples with the substitution rate *x* of 0.09–0.15, the presence of B-β-CsSnI_3_ (PDF2 file #80-2138) was observed. This β-phase is stable thermodynamically at higher temperatures of 78–152 °C but is present in the RS samples together with low-temperature B-γ-CsSnI_3_.

Table 1 shows the unit-cell parameters for the perovskite-related phase of Cs_2_SnI_6_, which were determined using the Le Bail method. The estimated unit-cell parameter *a* for the undoped Cs_2_SnI_6_ perovskite phase was found to be 11.6416 (8) Å, which is close to recently reported data (11.630 (10) Å, according to the PDF2 file 73–330). The evolution of the unit-cell parameter with increasing dopant content for the SS samples demonstrated a slight increase of the cell parameter *a* to 11.6426 (5) Å (*x* = 0.05). The unit-cell volume *V* changes only slightly. It changes the same way with an increase of gallium percentage in the charge (Table 1). It is important that the radius of Ga^3+^ is smaller than that of Sn^4+^ (0.62 Å vs. 0.69 Å), and only an insignificant decrease of the unit-cell parameter is expected if gallium(III) partially substitutes for tin(IV). The deficiency in the iodine sublattice could also influence cubic unit-cell parameters in the same manner. In contrast, substitution of Sn^4+^ ions with the larger cations of Sn^2+^ could act in a different way by increasing the unit-cell parameter and volume.

The unit-cell parameter *a* of the “double perovskite” Cs_2_SnI_6_ phase was estimated for the RS series to demonstrate its evolution with the substitution rate. The results are presented in Table 2. As soon as the samples include more than one phase of Ga-doped Cs_2_SnI_6_, these values are not characteristic for the substituted phases.

**Figure 2:**
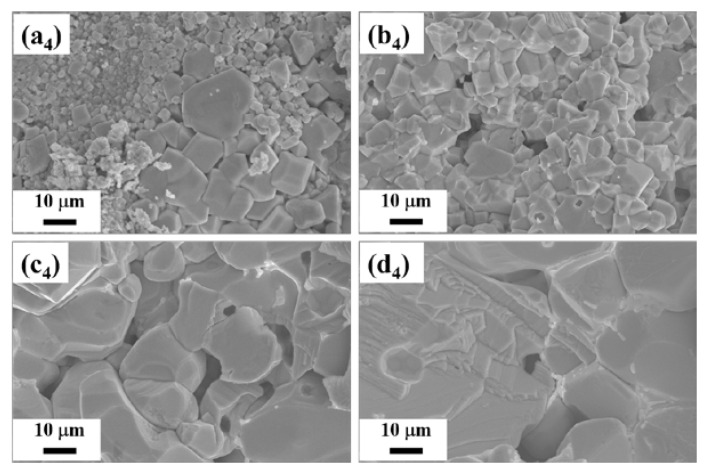
SEM images of fracture of the samples (**a_4_**) *x* = 0, (**b_4_**) *x* = 0.01, (**c_4_**) *x* = 0.03, and (**d_4_**) *x* = 0.05 (SS-series) after annealing (before grinding). All micrographs are taken at the same magnification.

To investigate the effect of gallium doping on the crystallization process of the Cs_2_SnI_6_ phase, the morphological analysis of the as-prepared SS samples (before grinding) was carried out with scanning electron microscopy (SEM). In order to correctly estimate the distribution of gallium over the volume of the sample, the annealed materials were divided into several parts so that the surface and the fracture (volume) could be explored (Figure S1). In the SEM images (Figure 2 and Figure S2), we can see that, on average, 100~200-micron spherical grains consist of crystallites of different sizes and shapes. Comparatively, Figure S2a4–d4 (*x* = 0, *x* = 0.01, *x* = 0.03, and *x* = 0.05) provides crystal size spreading based on the fracture top-view images. It is noticeable also that the characteristic grain size increased up to 50 μm with a gallium percentage in a melt, probably as a result of an excess of iodine in the ampoules. A similar effect was found recently for Cs_2_SnI_6_ and many other complex iodides [40]. For details, see also Figures S2–S8.

The EDX images and tables of the elemental composition of the samples show that gallium is not evenly distributed across the samples. A large amount of gallium is observed at the grain boundaries, which indicates the possibility of the formation of the CsGaI_4_ phase. Since CsGaI_4_ is an ionic liquid and remains in a liquid state at a temperature of 300 °C, it becomes an ion exchanger (or solvent) between precursors/Cs_2_SnI_6_, and this is the reason for the increase in the crystal size in doped samples. Gallium can also be seen in Cs_2_SnI_6_ crystallites (Figures S3 and S7), albeit in small quantities. We assume that gallium in the Cs_2_SnI_6_ structure does not replace tin but is present in the form of interstitial defects in octahedral vacancies (as shown in Figure 1a), so it is not distributed uniformly. Additionally, it should be noted that in the micrographs of backscattered electrons, a chemical contrast that would show the presence of two phases is not observed.

**Figure 3:**
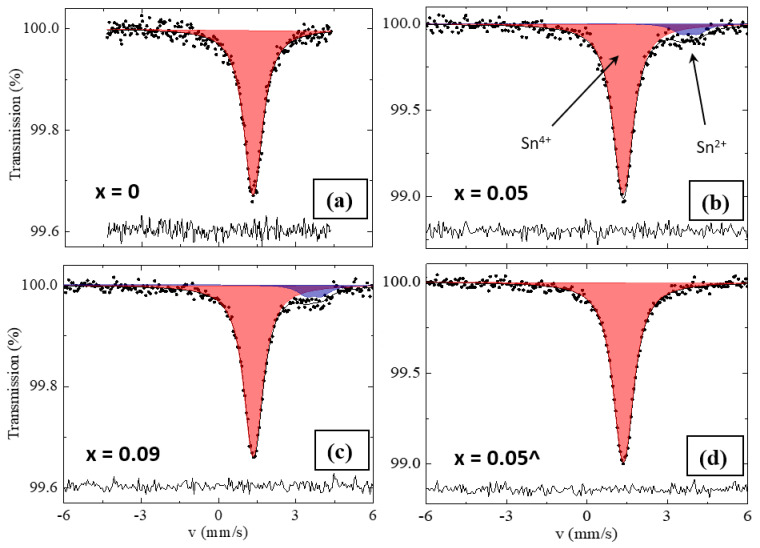
(**a**–**c**) Mössbauer spectra of the product of reduction of Cs_2_SnI_6_ with gallium melt with *x* = 0–0.09; (**d**) SS sample with *x* = 0.05^.

The ^119^Sn Mössbauer spectroscopy was used to analyze the oxidation state of tin atoms in both series of samples. Figure 3a–c shows characteristic ^119^Sn Mössbauer spectra for RS samples (*x* = 0; 0.05; 0.09) after 2 days of spectra collecting (Table 3). In the Mössbauer spectra of the RS series (*x* = 0.05 and 0.09), the presence of a major sensibly unresolved resonant subspectrum characteristic of Sn^4+^ (isomer shift *δ* ≈ 1.36 mm/s, quadrupole splitting ∆ ≈ 0.15 mm/s) and one minor doublet that corresponds to Sn^2+^ (isomer shift *δ* = 3.84 mm/s and *δ* = 3.72 mm/s for *x* = 0.05 and *x* = 0.09, respectively) are observed.

The major component can be attributed to the cubic Cs_2_SnI_6_ phase [39]. The isomer shift and profile of the spectrum for the double perovskite is further confirmed by the data for the SS sample (*x* = 0.05) given in Figure 3d. The hyperfine parameters of the phases are given in Table 3. The presence of Sn^2+^ ions within the sample of the SS series was not found. For the RS series, Mössbauer spectroscopy data do not contradict the results of the XRD phase composition analysis. Additionally, the experimental values of the hyperfine parameters correspond to previously reported data for the three presented individual phases [39,41]. According to Yamada et al. [42], minor quadrupole doublets correspond to the β-CsSnI_3_ phase. The doublet related to the B-γ-CsSnI_3_ phase, which has higher quadrupole splitting, is not found, and we assume that this is due to the small amount of this phase in the sample. An excess of the β-CsSnI_3_ phase in the RS sample with *x* = 0.05 could be a result of the concentration gradient of gallium in the sample.

To investigate the optical properties of the black-color materials, diffuse reflectance spectra were analyzed in the wavelength range of 200–2000 nm. The estimated optical band gap values for the samples are in the range of 1.23–1.35 eV and diminish with increasing the gallium percentage in the samples. The profiles of the collected absorption spectra correspond to direct-gap semiconductors [43], while the estimated optical *E_g_* values for the samples demonstrate larger Urbach energies for both RS and SS samples with the smaller concentration of gallium dopant. We assume that the decrease in the absorption edge intensity and its slight shift are due to a suppression of vacancies/intrinsic defects with a low formation energy and formation of in-gap states. If the pristine Cs_2_SnI_6_ phase is the *p*-type semiconductor, this phenomenon would contradict the theory. If the SS samples of Cs_2_Sn_1-x_Ga_x_I_6-x_ are n-conductive, as reported by most authors, the Ga^3+^ atoms will serve as electron traps or increase the e’-h˙ recombination probability.

**Figure 4:**
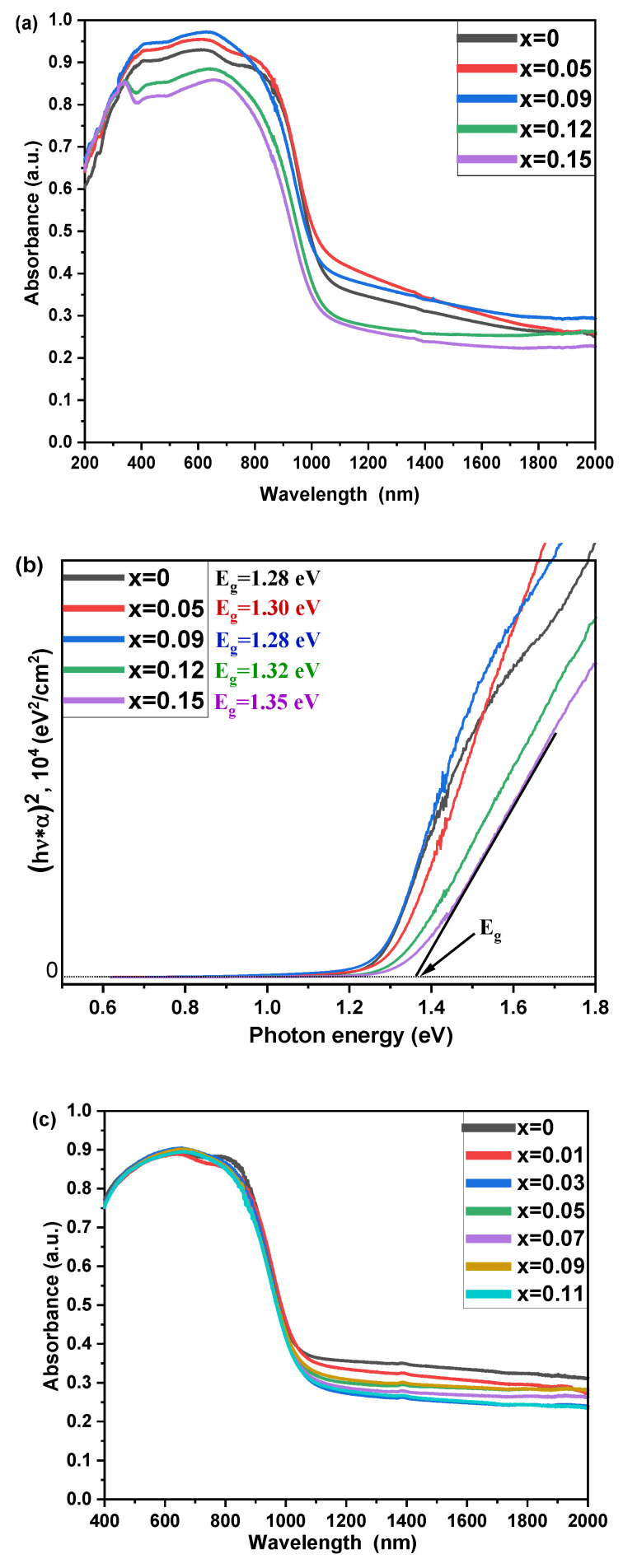

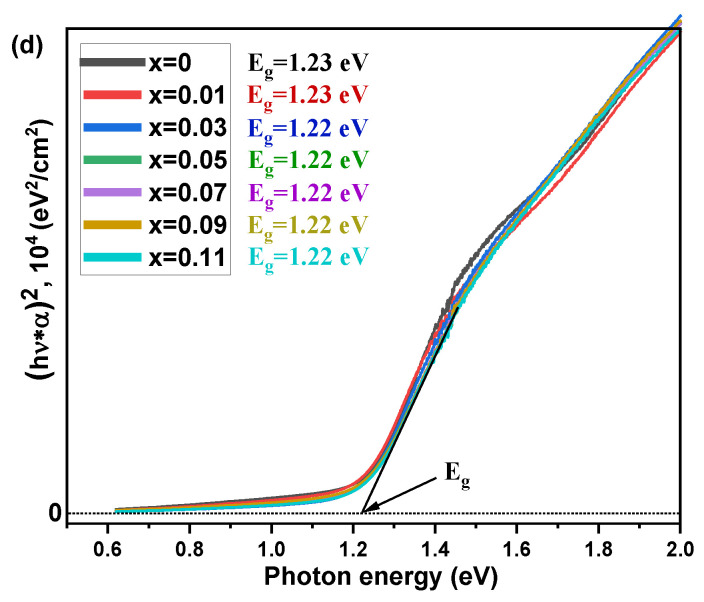
(**a**) Optical absorption spectra and (**b**) Tauc plots of the absorbance data for RS samples and (**c**,**d**) for SS samples, respectively.

For the RS series (Figure 4a,b), we observed a wider absorption edge than that of the SS series. Most likely, most of the spectra correspond to the superposition of subspectra of the SS phase of Cs_2_Sn_1-x_Ga_x_I_6-x_ (*E_g_* observed at 1.31–1.35 eV) and the black-color phase of γ-CsSnI_3_ (*E_g_* = 1.23 eV [8]). This correlates partly to the absorption spectra of these materials, revealing local maxima at ~630 nm for all samples (Figure 4a,c), with small discrepancies within the SS and RS series.

Thus, the reduction of Sn^4+^ to Sn^2+^ by gallium melt leads to the formation of perovskite phases, namely, the black γ-CsSnI_3_ genuine perovskite and β-CsSnI_3_ phase as an admixture. The latter is promising for light harvesting. It is remarkable also that Cs_2_SnI_6_ is a *p*-type semiconductor [22] and γ-CsSnI_3_ is an *n*-type semiconductor [7]. This fact makes the growth of the sandwich-like planar structures of Cs_2_SnI_6_ and γ-CsSnI_3_ attractive, aiming at the *p–n* heterojunction materials for the solid-state solar cells [44]. Thus, the composite made of Ga-doped Cs_2_SnI_6_ with γ-CsSnI_3_ and β-CsSnI_3_ perovskites seems to be a model for further investigation of corresponding cesium iodostannates (II, IV) composite films.

## 4. Conclusions

Our study improves the understanding of the double-perovskite Cs_2_SnI_6_ perspective as a perovskite solar-cell compound. According to the XRD data, no iodogallates were found in the samples for the *x* < 0.05 compositions, and so the formation of the substitutional solid solution of the double-perovskite Cs_2_Sn_1-x_Ga_x_I_6-x_ up to 5 at.% of Ga is still under discussion. On the other hand, the interaction of the tin-based double-perovskite Cs_2_SnX_6_ with metal gallium leads to the formation of a new light-harvesting composite compound and a hole- or electron-transport material with improved grain boundaries and appropriate conductivities of charge carriers. It is remarkable also that the presence of Sn^2+^ cations in the double-perovskite structure is still under discussion due to the easy segregation of tin(II) in a form of individual phases of β-CsSnI_3_ and γ-CsSnI_3_. These results suggest that it is possible to optimize the crystal quality and optoelectronic properties by doping the perovskite structures (ABX_3_, A_2_BX_6_, A_3_B_2×9_) with relevant heterovalent cations. We expect that this research will be a significant step for obtaining “less toxic” light-absorber materials with improved characteristics.

## Figures and Tables

**Table 1:** Calculated cell parameters for the Cs_2_Sn_1−x_Ga_x_I_6−x_ samples (SS series).

*x*	Phase	a, Å	Cell Volume, Å^3^	Rp, %	wRp, %	GOF (χ^2^)
0	Cs_2_SnI_6_	11.6416 (8)	1577.75 (11)	7.55	10.63	1.62
0.01	Cs_2_SnI_6_	11.6418 (9)	1577.85 (13)	7.37	10.42	1.50
0.03	Cs_2_SnI_6_	11.6411 (5)	1577.58 (7)	5.59	8.69	1.31
0.05	Cs_2_SnI_6_	11.6426 (5)	1578.17 (7)	6.11	9.26	1.3
0.07	Cs_2_SnI_6_	11.6411 (3)	1577.57 (4)	7.14	10.54	1.29
0.09	Cs_2_SnI_6_	11.6398 (7)	1577.04 (9)	9.48	14.14	1.19
0.11	Cs_2_SnI_6_	11.6407 (9)	1577.41 (12)	9.03	13.35	1.15

**Table 2:** Calculated cell parameters for the Cs_2_SnI_6_ samples reduced by metallic gallium (RS series).

*x*	Phase	a, Å	Cell Volume, Å^3^	Rp, %	wRp, %	GOF (χ^2^)
0	Cs_2_SnI_6_	11.6458 (9)	1579.450 (13)	9.3	13.19	1.25
0.05	Cs_2_SnI_6_	11.6438 (3)	1578.654 (2)	9.8	14.17	1.17
	CsSnI_3_	8.7252 (1)	477.137 (7)			
0.09	Cs_2_SnI_6_	11.6482 (1)	1580.43 (2)	10.16	14.8	1.22
	CsSnI_3_	8.7339 (8)	477.29 (6)			
0.12	Cs_2_SnI_6_	11.647 (1)	1580.26 (2)	10.64	15.01	1.20
	CsSnI_3_	8.6995 (2)	482.23 (8)			
0.15	Cs_2_SnI_6_	11.664 (2)	1587.18 (2)	11.81	16.43	1.27
	CsSnI_3_	8.6016 (2)	461.24 (10)			

**Table 3:** Hyperfine parameters of the ^119^Sn Mössbauer spectra of RS and SS samples with different gallium content (*x*) at RT.

Series	*x*	Tin Type	δ (mm/s)	∆ (mm/s)	W (mm/s)	A (%)
RS	0.00	Sn^4+^	1.36 (1)	0.15 (4)	0.85 *	100
	0.05	Sn^4+^	1.35 (1)	0.18 (3)	0.85 *	91.4 (9)
		Sn^2+^	3.84 (8)	0.31 (7)	0.85 *	8.6 (9)
	0.09	Sn^4+^	1.36 (1)	0.17 (3)	0.85 *	90.9 (7)
		Sn^2+^	3.72 (7)	0.30 (6)	0.85 *	9.1 (7)
SS	0.05^	Sn^4+^	1.37 (1)	0.22 (2)	0.85 *	100

* indicates values are fixed; W is the full width at the half height; A is a percent area of subspectrum.

## Data Availability

Not applicable.

## Supplementary Materials

The following supporting information can be downloaded at: https://www.mdpi.com/article/10.3390/nano13030427/s1. Table S1: Composition of Cs_2_Sn_1-x_Ga_x_I_6-x_ solid-solution samples (SS series); Table S2: Composition of samples for the reduction of Cs_2_SnI_6_ by metallic gallium (RS); Figure S1: Optical photographs of a piece of compound after synthesis (before grinding) for SEM and EDX measurements (an example of the SS series); Figure S2: SEM images of surface and fracture of all SS series samples; Figure S3: Secondary electron image (a) and (b) backscattered electrons image (with chemical contrast) of fracture and (c) element distribution map of the *x* = 0.03 SS sample; Figure S4: EDX results (including SEM images and elements table) of fracture of the *x* = 0.03 SS sample; Figure S5: Backscattered electrons (with chemical contrast) images of surface (a) and (b) fracture of the *x* = 0.05 SS sample; Figure S6: EDX results (including SEM images and elements table) of fracture of the *x* = 0.05 SS sample; Figure S7: Secondary electrons image (a) and (b) backscattered electrons image (with chemical contrast) of fracture and (c) element distribution map of the *x* = 0.11 SS sample; Figure S8: EDX results of surface (a) and (b) fracture of the *x* = 0.11 SS sample and (c) backscattered electrons image.

## References

[1] Manser J.S., Christians J.A., Kamat P.V. (2016). Intriguing optoelectronic properties of metal halide perovskites. Chem. Rev..

[2] Mitzi D.B. (2007). Synthesis, structure, and properties of organic-inorganic Perovskites and related materials. Prog. Inorg. Chem..

[3] Min H., Lee D.Y., Kim J., Kim G., Lee K.S., Kim J., Paik M.J., Kim Y.K., Kim K.S., Kim M.G. (2021). Perovskite solar cells with atomically coherent interlayers on SnO_2_ electrodes. Nature.

[4] Penconi M., Bianchi G., Nitti A., Savoini A., Carbonera C., Pasini D., Po R., Luzzati S. (2021). A donor polymer with a good compromise between efficiency and sustainability for organic solar cells. Adv. Energy Sustain. Res..

[5] Na H.-J., Lee S.-E., Lee E.G., Lee J.H., Gong Y.J., Kim H., Cho N.-K., Kim Y.S. (2021). Nanometer-thick Cs_2_SnI_6_ perovskite—Polyethylene glycol dimethacrylate composite films for highly stable broad-band photodetectors. ACS Appl. Nano Mater..

[6] Matthews P.D., Lewis D.J., O’Brien P. (2017). Updating the road map to metal-halide perovskites for photovoltaics. J. Mater. Chem. A.

[7] Green M.A., Emery K., Hishikawa Y., Warta W., Dunlop E.D. (2016). Solar cell efficiency tables. Prog. Photovolt. Res. Appl..

[8] Chung I., Lee B., He J., Chang R.P.H., Kanatzidis M.G. (2012). All-solid-state dye-sensitized solar cells with high efficiency. Nature.

[9] Yu C., Chen Z., Wang J.J., Pfenninger W., Vockic N., Kenney J.T., Shum K. (2011). Temperature dependence of the band gap of perovskite semiconductor compound CsSnI_3_. J. Appl. Phys..

[10] Chen Z., Yu C., Shum K., Wang J.J., Pfenninger W., Vockic N., Midgley J., Kenney J.T. (2012). Photoluminescence study of pol-ycrystalline CsSnI_3_ thin films: Determination of exciton binding energy. J. Lumin..

[11] Kumar M.H., Dharani S., Leong W.L., Boix P.P., Prabhakar R.R., Baikie T., Shi C., Ding H., Ramesh R., Asta M. (2014). Lead-free halide perovskite solar cells with high photocurrents realized through vacancy modulation. Adv. Mater. Lett..

[12] Stoumpos C.C., Malliakas C.D., Kanatzidis M.G. (2013). Semiconducting tin and lead iodide perovskites with organic cations: Phase transitions, high mobilities, and near-infrared photoluminescent properties. Inorg. Chem..

[13] Yamada K., Funabiki S., Horimoto H., Matsui T., Okuda T., Ichiba S. (1991). Structural phase transitions of the polymorphs of CsSnI_3_ by means of rietveld analysis of the X-ray diffraction. Chem. Lett..

[14] Scaife D.E., Weller P.F., Fisher W.G. (1974). Crystal preparation and properties of cesium tin(II) trihalides. J. Solid State Chem..

[15] Chung I., Song J.-H., Im J., Androulakis J., Malliakas C.D., Li H., Freeman A.J., Kenney J.T., Kanatzidis M.G. (2012). CsSnI_3_: Semiconductor or metal? High electrical conductivity and strong near-infrared photoluminescence from a single material. J. Am. Chem. Soc..

[16] Marshall K.P., Walker M., Walton R.I., Hatton R.A. (2017). Elucidating the role of the hole-extracting electrode on the stability and efficiency of inverted CsSnI_3_/C_60_ perovskite photovoltaics. J. Mater. Chem. A.

[17] Wijesekara A., Varagnolo S., Dabera G.D.M.R., Marshall K.P., Pereira H.J., Hatton R.A. (2018). Assessing the suitability of copper thiocyanate as a hole-transport layer in inverted CsSnI_3_ perovskite photovoltaics. Sci. Rep..

[18] Huang L., Lambrecht W.R.L. (2013). Electronic band structure, phonons, and exciton binding energies of halide perovskites CsSnCl_3_, CsSnBr_3_, and CsSnI_3_. Phys. Rev. B.

[19] Lora da Silva E., Skelton J.M., Parker S.C., Walsh A. (2015). Phase stability and transformations in the halide perovskite CsSnI_3_. Phys. Rev. B.

[20] Zhang J., Yu C., Wang L., Lili W., Ren Y., Shum K. (2014). Energy barrier at the N719-dye/CsSnI_3_ interface for photogenerated holes in dye-sensitized solar cells. Sci. Rep..

[21] Chen Z., Wang J.J., Ren Y., Yu C., Shum K. (2012). Schottky solar cells based on CsSnI_3_ thin-films. Appl. Phys. Lett..

[22] Lee B., Stoumpos C.C., Zhou N., Hao F., Malliakas C., Yeh C.-Y., Marks T.J., Kanatzidis M.G., Chang R.P.H. (2014). Air-stable molecular semiconducting iodosalts for solar cell applications: Cs_2_SnI_6_ as a hole conductor. J. Am. Chem. Soc..

[23] Qiu X., Jiang Y., Zhang H., Qiu Z., Yuan S., Wang P., Cao B. (2016). Lead-free mesoscopic Cs_2_SnI_6_perovskite solar cells using different nanostructured ZnO nanorods as electron transport layers. Phys. Status Solidi (RRL) Rapid Res. Lett..

[24] Chander N., Chandrasekhar P.S., Komarala V.K. (2014). Solid state plasmonic dye sensitized solar cells based on solution processed perovskite CsSnI_3_ as the hole transporter. RSC Adv..

[25] Werker W. (1939). Die krystallstruktur des Rb_2_SnI_6_ und Cs_2_SnI_6_. Recl. Trav. Chim. Pays-Bas.

[26] Kontos A.G., Kaltzoglou A., Siranidi E., Palles D., Angeli G.K., Arfanis M.K., Psycharis V., Raptis Y.S., Kamitsos E.I., Trikalitis P.N. (2016). Structural stability, vibrational properties, and photolumines-cence in CsSnI_3_ perovskite upon the addition of SnF_2_. Inorg. Chem..

[27] Saparov B., Sun J., Meng W., Xiao Z., Duan H., Gunawan O., Shin D., Hill I.G., Yan Y., Mitzi D.B. (2016). Thin-film deposition and characterization of a Sn-deficient perovskite derivative Cs_2_SnI_6_. Chem. Mater..

[28] Xiao Z., Lei H., Zhang X., Zhou Y., Hosono H., Kamiya T. (2015). Ligand-hole in [SnI_6_] unit and origin of band gap in photo-voltaic perovskite variant Cs_2_SnI_6_. Bull. Chem. Soc. Jpn..

[29] Xiao Z., Zhou Y., Hosono H., Kamiya T. (2015). Intrinsic defects in photovoltaic perovskite variant Cs_2_SnI_6_. Phys. Chem. Chem. Phys..

[30] Ju M.G., Chen M., Zhou Y., Garces H., Dai J., Padture N.P., Zeng X.C. (2018). Earth-abundant nontoxic titanium(IV)-based va-cancy-ordered double perovskite halides with tunable 1.0 to 1.8 eV bandgaps for photovoltaic applications. ACS Energy Lett..

[31] Wang G., Wang D., Shi X. (2015). Electronic structure and optical properties of Cs_2_AẊ_2_X_4_ (A = Ge, Sn, Pb; Ẋ, X = Cl, Br, I). AIP Adv..

[32] Zhang P., Yang J., Wei S.-H. (2017). Manipulation of cation combinations and configurations of halide double perovskites for solar cell absorbers. J. Mater. Chem. A.

[33] Lee B., Krenselewski A., Baik S.I., Seidman D.N., Chang R.P.H. (2017). Solution processing of air-stable molecular semiconducting iodosalts, Cs_2_SnI_6_-xBrx, for potential solar cell application. Sustain. Energy Fuels.

[34] Umedov S.T., Grigorieva A.V., Lepnev L.S., Knotko A.V., Nakabayashi K., Ohkoshi S.-I., Shevelkov A.V. (2020). Indium doping of lead-free perovskite Cs_2_SnI_6_. Front. Chem..

[35] Ke J.C.R., Lewis D.J., Walton A.S., Spencer B.F., O’Brien P., Thomas A.G., Flavell W.R. (2018). Ambient-air-stable inorganic Cs_2_SnI_6_ double perovskite thin films via aerosol-assisted chemical vapour deposition. J. Mater. Chem. A.

[36] Wu J., Zhao Z., Zhou Y. (2022). The optoelectronic properties improvement of double perovskites Cs_2_SnI_6_ by anionic doping (F^−^). Sci. Rep..

[37] Matsnev M.E., Rusakov V.S. (2012). SpectrRelax: An application for mossbauer spectra modeling and fitting. AIP Conf. Proc..

[38] Matsnev M.E., Rusakov V.S. (2014). Study of spatial spin-modulated structures by Mössbauer spectroscopy using SpectrRelax. AIP Conf. Proc..

[39] Tudela D., Sánchez-Herencia A.J., Díaz M., Fernández-Ruiz R., Menéndez N., Tornero J.D. (1999). Mössbauer spectra of tin(IV) iodide complexes. Dalton Trans..

[40] Ullah S., Yang P., Wang J., Liu L., Li Y., Zafar Z., Yang S.E., Xia T., Guo H., Chen Y. (2021). The fabrication of lead-free Cs_2_SnI_6_ perovskite films using iodine-rich strategy for optoelectronic applications. Phys. Status Solidi A.

[41] Dalpian G.M., Liu Q., Stoumpos C.C., Douvalis A.P., Balasubramanian M., Kanatzidis M.G., Zunger A. (2017). Changes in charge density vs. changes in formal oxidation states: The case of Sn halide perovskites and their ordered vacancy analogues. Phys. Rev. Mater..

[42] Yamada K., Matsui T., Tsuritani T., Okuda T., Ichiba S. (1990). 127I-NQR, 119Sn Mössbauer effect, and electrical conductivity of MSnI_3_ (M = K, NH_4_, Rb, Cs, and CH_3_NH_3_). Z. Nat..

[43] Qiu X., Cao B., Yuan S., Chen X., Qiu Z., Jiang Y., Ye Q., Wang H., Zeng H., Liu J. (2017). From unstable CsSnI_3_ to air-stable Cs_2_SnI_6_: A lead-free perovskite solar cell light absorber with bandgap of 1.48 eV and high absorption coefficient. Sol. Energy Mater. Sol. Cells.

[44] Zhang J., Li S., Yang P., Liu W., Liao Y. (2018). Enhanced stability of lead-free perovskite heterojunction for photovoltaic applications. J. Mater. Sci..

