# Supplementary material for: Controlled Reduction of Sn4+ in the Complex Iodide Cs2SnI6 with Metallic Gallium
Source: Nanomaterials (Basel). 2023 Jan 20;13(3):427. doi: 10.3390/nano13030427 (PMC9919842; doi:10.3390/nano13030427)
Supplement: Supplementary file 1 [file nanomaterials-13-00427-s001.zip › nanomaterials-2153692-supplementary.pdf]

Supplementary Materials

Controlled Reduction of Sn^{4+} in the Complex Iodide Cs_2SnI_6 with Metallic Gallium

Shodruz T. Umedov [1], Anastasia V. Grigorieva [1,2,*], Alexey V. Sobolev [2,3], Alexander V. Knotko [1,2], Leonid S. Lepnev [4], Efim A. Kolesnikov [1], Dmitri O. Charkin [2] and Andrei V. Shevelkov [2]

[1] Department of Materials Science, Lomonosov Moscow State University, Leninskie Gory 1/73, 119991 Moscow, Russia
[2] Department of Chemistry, Lomonosov Moscow State University, Leninskie Gory 1/3, 119991 Moscow, Russia
[3] Department of Chemistry, MSU-BIT University, Shenzhen 517182, China
[4] Lebedev Physical Institute of the Russian Academy of Sciences, Leninskiy Prospect 53, 119333 Moscow, Russia; llepn@mail.ru
* Correspondence: anastasia@inorg.chem.msu.ru; Tel.: +7-(495)-939-4609; Fax: +7-(495)-939-0998

The experimental details concerning the composition of the powder mixture before annealing.

Table S1. Composition of $Cs_2Sn_{1-x}Ga_xI_{6-x}$ solid solution samples (SS series).

№	*x*	m (CsI), g	m (SnI_4), g	m (Ga), g	m (I), g
1	0	0.4534	0.5466	0	0
2	0.01	0.4536	0.5413	0.0006	0.0044
3	0.03	0.4540	0.5308	0.0018	0.0133
4	0.05	0.4544	0.5203	0.0030	0.0222
5	0.07	0.4548	0.5098	0.0042	0.0311
6	0.09	0.4551	0.4992	0.0055	0.0400
7	0.11	0.4555	0.4887	0.0067	0.0489

Table S2. Composition of samples for the reduction of Cs_2SnI_6 by metallic gallium (RS).

№	*x*	m (CsI), g	m (SnI_4), g	m (Ga), g
1	0	0.4534	0.5466	0
2	0.01	0.4579	0.5409	0.0012
3	0.05	0.4766	0.5170	0.0064
4	0.09	0.4969	0.4911	0.0120
5	0.12	0.5133	0.4702	0.0165
6	0.15	0.5308	0.4479	0.0214

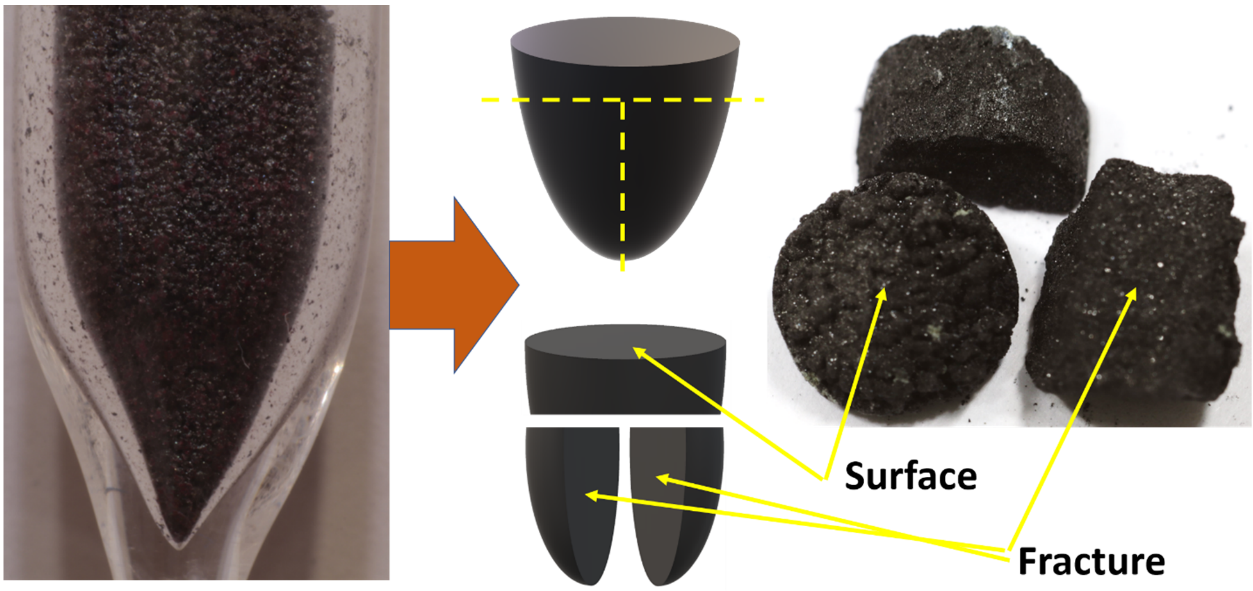

Figure S1. Optical photographs of a piece of compounds after synthesis (before grinding) for SEM and EDS measurements (example of SS series).

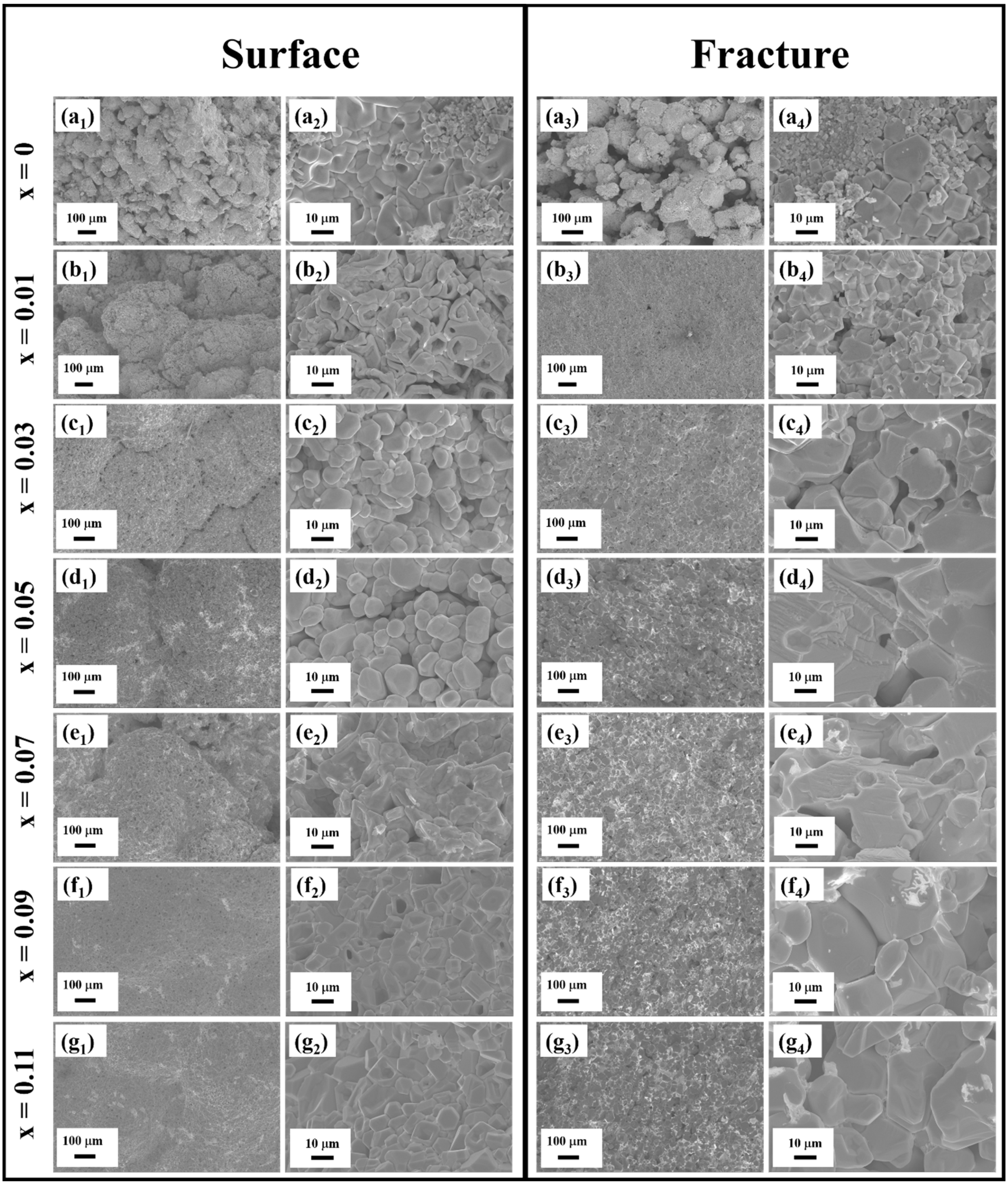

Figure S2. SEM images of surface and fracture of all SS series samples.

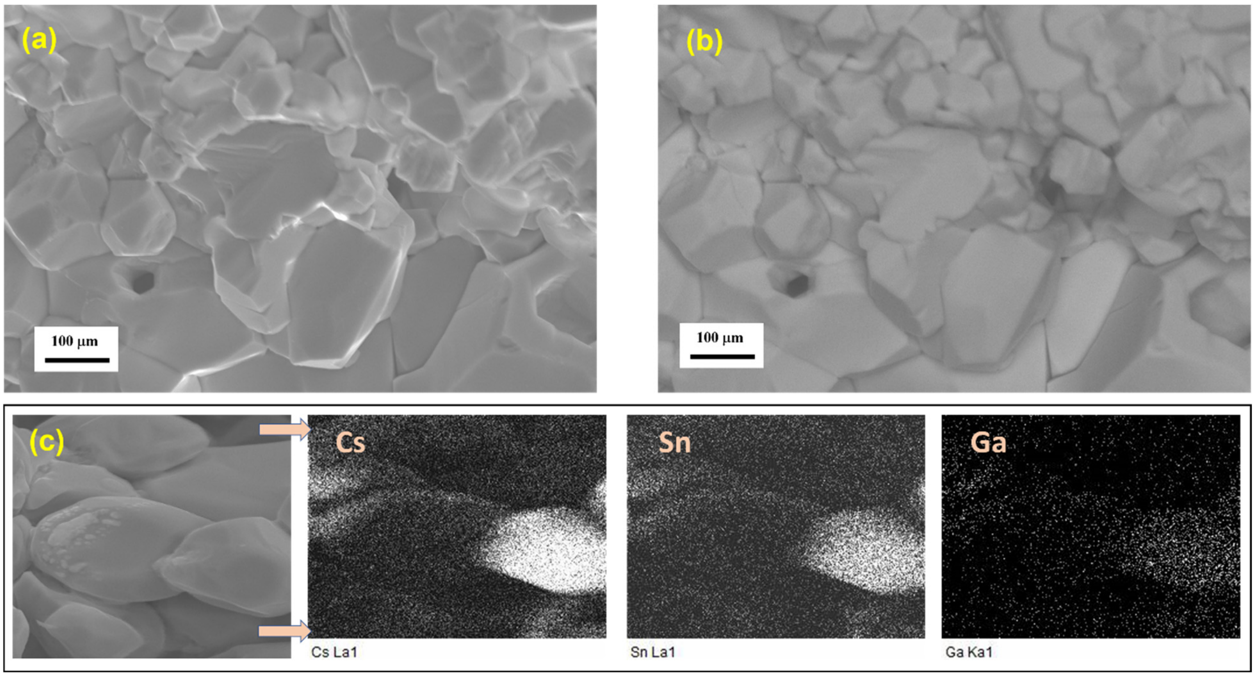

Figure S3. Secondary electron image (a) and (b) backscattered electrons image (chemical contrast) of fracture and (c) element distribution map of $x = 0.03$ SS sample.

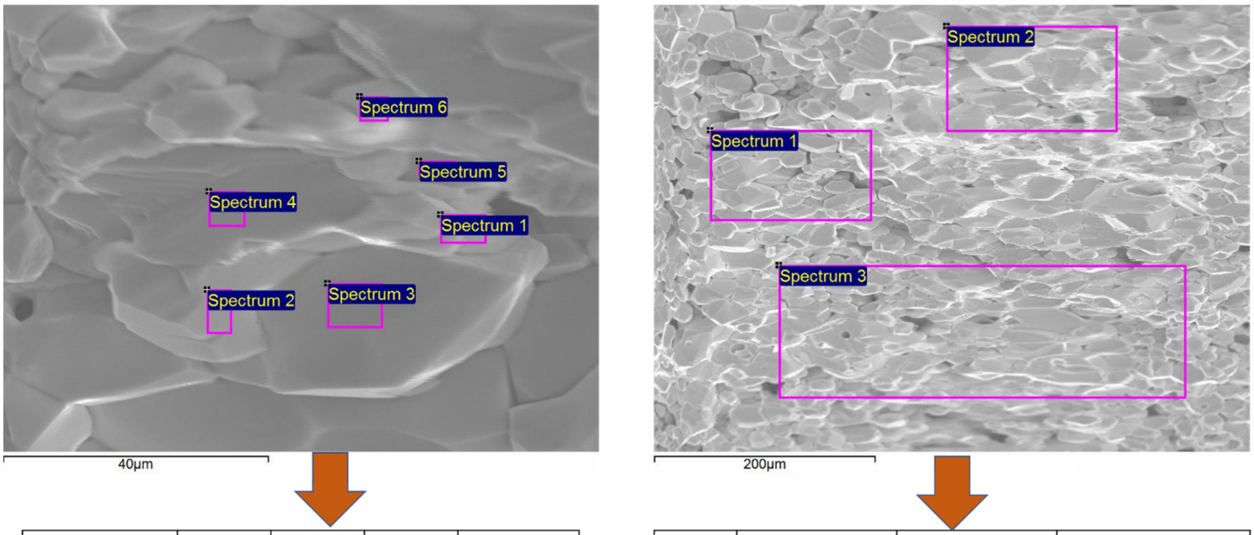

Spectrum	Ga	Sn	I	Cs
1	5.43	7.79	67.64	19.15
2	0.43	11.39	67.66	20.52
3	-0.1	11.51	67.75	20.84
4	0.69	11.23	67.5	20.58
5	9.96	4.26	68.53	17.26
6	10.04	4.12	68.49	17.34

	Spectrum 1	Spectrum 2	Spectrum 3
Ga	0.42	1.31	-0.31
Sn	11.14	10.51	11.58
I	68.1	67.84	68.25
Cs	20.35	20.35	20.48

Figure S4. EDX results (SEM images and elements table) of fracture of $x = 0.03$ SS sample.

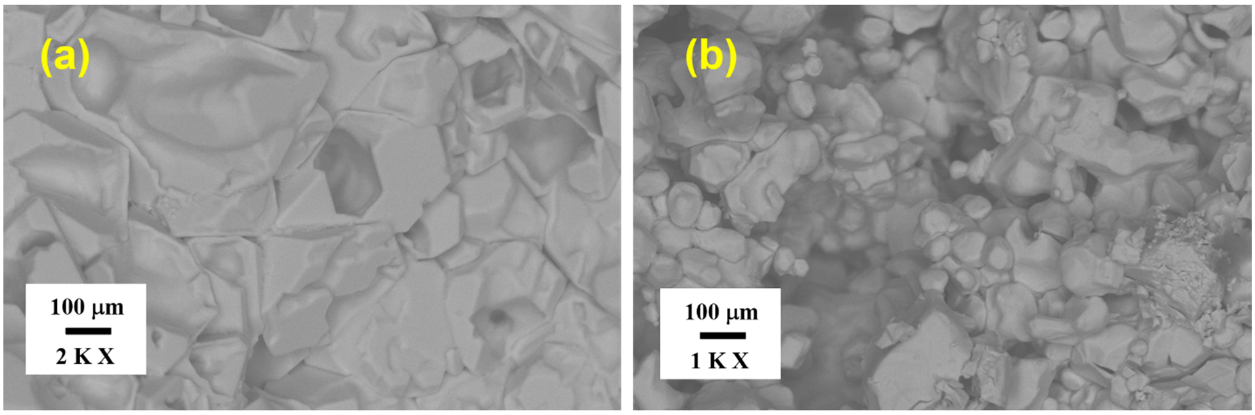

Figure S5. Backscattered electrons (chemical contrast) images of surface (a) and (b) fracture of x = 0.05 SS sample.

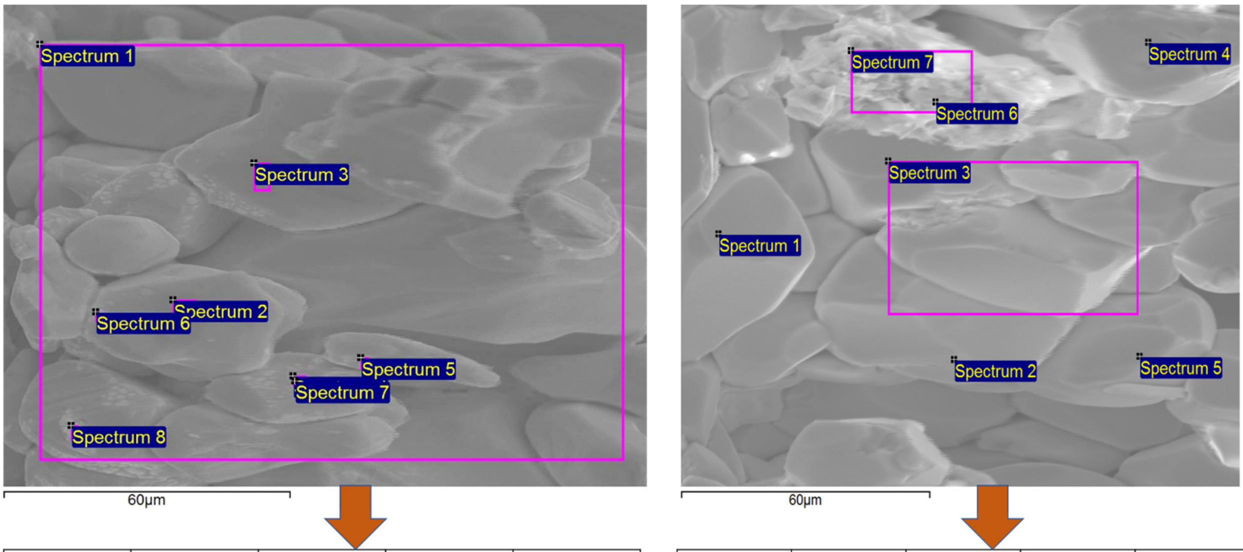

Spectrum	Ga	Sn	I	Cs
1	-0.1	11.2	68.28	20.63
2	0.12	11.42	67.68	20.78
3	0.26	11.62	67.36	20.75
4	0.02	9.96	69.72	20.3
5	0.03	10.69	68.19	21.09
6	0.22	11	68.44	20.34
7	0.08	10.2	68.4	21.33
8	1.27	9.78	67.23	21.71

Spectrum	Ga	Sn	I	Cs
1	0.77	8.51	71.53	19.19
2	1.06	8.37	70.92	19.65
3	0.7	10	68.51	20.79
4	0.18	10.5	67.54	21.78
5	0.18	9.38	69.32	21.11
6	10.58	1.52	73.11	14.78
7	9.09	1.55	71.57	17.79

Figure S6. EDX results (SEM images and elements table) of fracture of x = 0.05 SS sample.

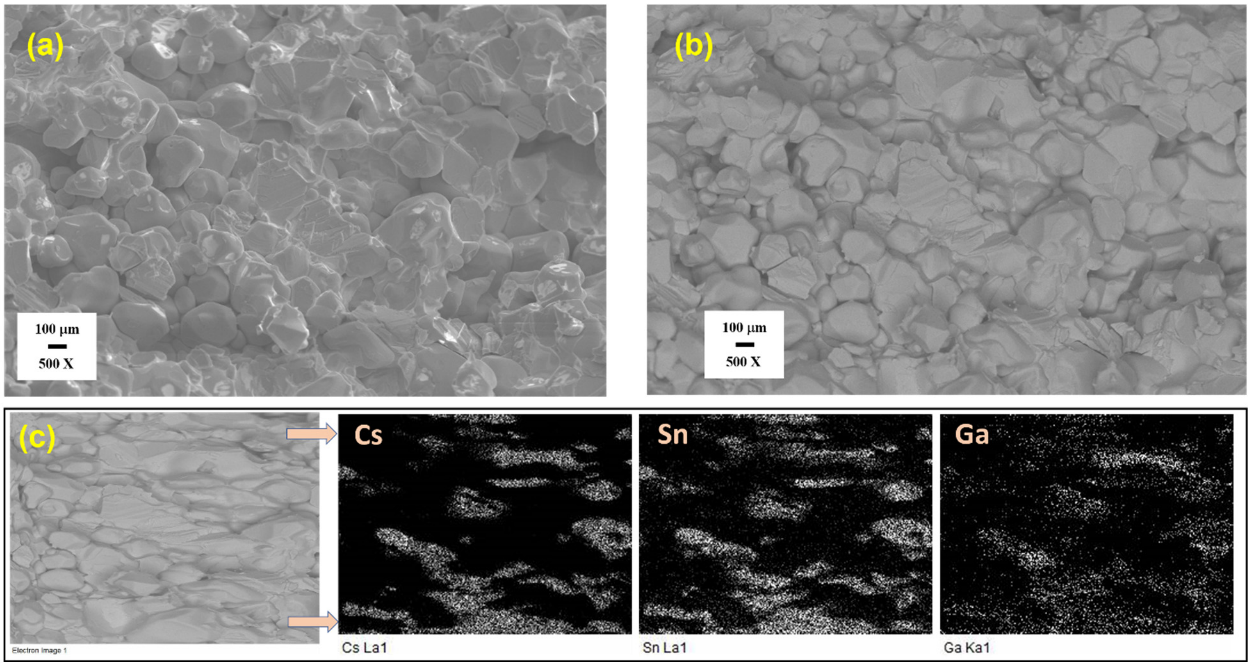

Figure S7. Secondary electrons image (a) and (b) backscattered electrons image (chemical contrast) of fracture and (c) element distribution map of x = 0.11 SS sample. (c) backscattered electrons image.

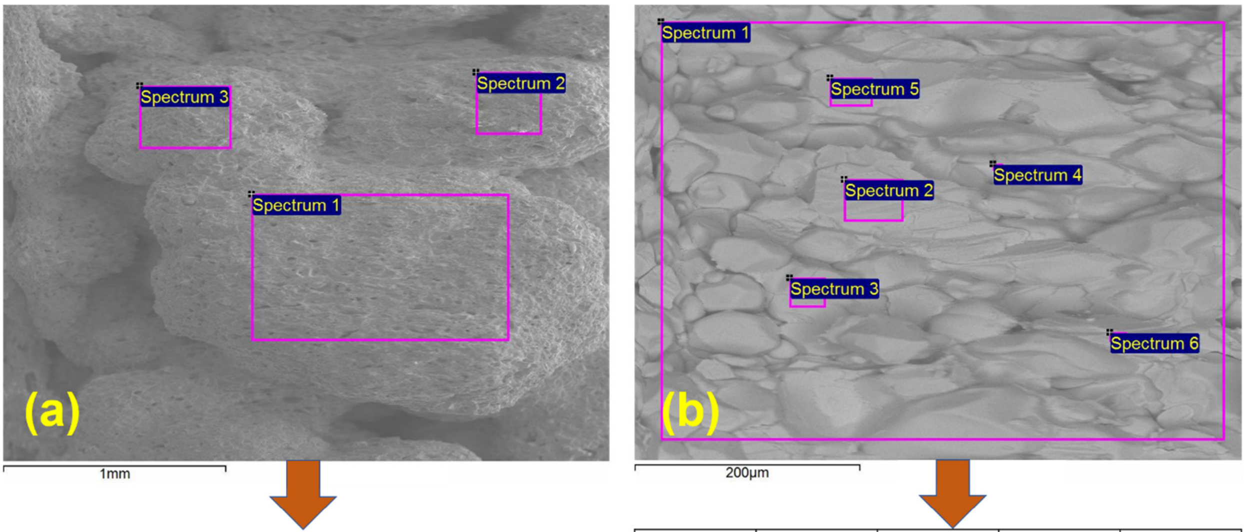

Spectrum	1	2	3
Ga	0,3	1,13	0,89
Sn	10,62	10,44	10,72
I	69,16	67,94	68,09
Cs	19,92	20,5	20,3

Spectrum	Ga	Sn	I	Cs
1	4,87	7,84	67,68	19,61
2	0,96	10,32	69,06	19,65
3	8,35	5,6	67,57	18,48
5	1,05	9,87	69,85	19,23
6	0,32	10,67	68,99	20,03

Figure S8. EDX results of surface (a) and (b) fracture of x = 0.11 SS sample. (b) backscattered electrons image.
